# Olfactory receptors in macrophages and inflammation

**DOI:** 10.3389/fimmu.2022.1029244

**Published:** 2022-10-13

**Authors:** Marco Orecchioni, Hiroaki Matsunami, Klaus Ley

**Affiliations:** ^1^ Department of Inflammation Biology, La Jolla Institute for Immunology, La Jolla, CA, United States; ^2^ Molecular Genetics and Microbiology, Duke University School of Medicine, Durham, NC, United States; ^3^ Immunology Center of Georgia, Augusta University, Augusta, GA, United States

**Keywords:** olfactory receptors, GPCRs, monocytes, macrophages, inflammation

## Abstract

Olfactory receptors (ORs) that bind odorous ligands are the largest family of G-protein-coupled receptors. In the olfactory epithelium, approximately 400 and 1,100 members are expressed in humans and mice, respectively. Growing evidence suggests the extranasal functions of ORs. Here, we review OR expression and function in macrophages, specialized innate immune cells involved in the detection, phagocytosis, and destruction of cellular debris and pathogens as well as the initiation of inflammatory responses. RNA sequencing data in mice suggest that up to 580 ORs may be expressed in macrophages. Macrophage OR expression is increased after treatment with the Toll-like receptor 4 ligand lipopolysaccharide, which also induces the transcription of inflammasome components. Triggering human OR6A2 or its mouse orthologue Olfr2 with their cognate ligand octanal induces inflammasome assembly and the secretion of IL-1β, which exacerbates atherosclerosis. Octanal is positively correlated with blood lipids like low-density lipoprotein –cholesterol in humans. Another OR, Olfr78, is activated by lactate, which promotes the generation of tumor-associated macrophages that dampen the immune response and promote tumor progression. Olfactory receptors in macrophages are a rich source of untapped opportunity for modulating inflammation. It is not known which of the many ORs expressed in macrophages promote or modulate inflammation. Progress in this area also requires deorphanizing more ORs and determining the sources of their ligands.

## Introduction

Olfactory receptors (ORs) were initially described in 1991 by Linda Buck and Richard Axel (1). In their initial observation, they suggested that this new family of receptors was restricted to the olfactory epithelium (OE) of the nose (1). These G-protein-coupled receptors (GPCRs) are activated by volatile chemical compounds also known as odorants (2). ORs in humans are proposed to be able to discriminate between anywhere from 10,000 to more than one trillion different smells (3).

However, OR expression is not limited to the nasal epithelium. ORs have been found in many different cell types and organs (4–7). In some cases, only messenger Ribonucleic Acid (mRNA) expression was reported, with unclear functionality. Traditionally, “ectopically” expressed ORs have been seen as possible expression artifacts. Often, they were deemed to exist without any real functional significance (3). Nowadays, “ectopic” ORs have been reported to exert important regulatory functions in homeostasis and disease statuses such as atherosclerosis, cancer growth, triglyceride metabolism, hepatic lipid accumulation, and obesity (8, 9). Various ORs respond to ligands including specific diet-related compounds, gut microbiota–derived metabolites, and oxidative stress. Thus, we propose to use the qualifier “extranasal” rather than “ectopic” to describe ORs in other organs, tissues, and cells. ORs require many accessory and signaling molecules to be functional. In the OE, ORs couple to the olfactory G protein alpha subunit (Golf), leading to the activation of adenylyl cyclase 3 (Adcy3) and the generation of cyclic adenosine monohosphate (cAMP) followed by the activation of cyclic nucleotide–gated (CNGs) ion channels and a subsequent Ca^2+^ flux (10).

OR sequences are found in all vertebrate species, being one of the most ancient sensory systems in animals, and are crucial for animal survival, procreation, and evolution (11, 12). Intriguingly, ORs were found expressed also in macrophages in both mice and humans (13–15). Macrophages are thought to be the primordial immune cells in vertebrates and non-vertebrates (16, 17);. A common hypothesis suggests that immune cells, especially phagocytes, share a common origin with the neuroendocrine system (18). Thus, macrophages are known to be influenced by neuroendocrine control; similarly, macrophages can influence the neuroendocrine axes, especially resident-like macrophages (18).

Macrophage origin can be broadly divided into tissue-resident macrophages and monocyte-derived macrophages. Resident macrophages derive from CX3CR1^+^ embryonic precursors that seed tissues pre- or perinatally, generating macrophages that proliferate locally and are long-lived (19). Tissue-resident macrophages can also have a postnatal contribution from bone marrow–derived monocytes that colonize the tissues after birth (20, 21).

Each tissue and organ has its own particular composition of embryonically derived and adult-derived macrophage subsets (22). During inflammation and damage, bone marrow–derived monocytes can populate the inflamed tissue, generating dendritic cells (DCs) and macrophages that impact the initiation, progression, and resolution of inflammation (23). Macrophages can have proinflammatory or anti-inflammatory features, defined often as classical M1 (proinflammatory) or alternative M2 (anti-inflammatory) polarization. This broad classification was defined mostly *in vitro* based on the specific features of Interferon gamma (IFNγ)-activated macrophages (M1) or IL-4-activated macrophages (M2) (24). *In vivo* macrophages acquire different shades of the activation status and functions (24).

Monocytes are conserved in all vertebrates, representing 4%–10% of all leukocytes (21). Monocytes are continuously generated in the bone marrow from hematopoietic stem cell precursors and are broadly defined based on the differential expression of specific markers such as LY6C^hi^ (classical) and LY6C^low^ (non-classical) in mice and CD14^+^ (classical) and CD14^low^CD16^+^ (non-classical) in humans. More monocyte subsets are being defined, suggesting a high heterogeneity of monocyte populations and functions (25).

Monocytes and macrophages have a highly specialized sensory system of pattern-recognition receptors, GPCRs, and tyrosine kinase receptors (26). Among the GPCRs, ORs can “smell” metabolites and allow macrophages to respond to their environment. This response can, among others, mediate the activation of the inflammasome (13) that is also highly conserved among vertebrates (27). The activation of the NLRP3 inflammasome requires a priming signal (signal 1), often mediated by the Toll-like receptor 4 (TLR4), followed by a secondary signal (signal 2) that promotes the interaction of NLRP3 subunits with the pyrin domain of apoptosis-associated speck-like protein containing a caspase recruitment domain (ASC) to initiate inflammasome assembly, which results in IL-1β processing by caspase 1 and can induce programmed inflammatory cell death (28). This process is good for defenses against opportunistic pathogens; however, if not resolved, it can be deleterious and drive chronic inflammation and the progression of several inflammatory diseases (13, 29).

In this review, we summarize recent discoveries highlighting the expression and function of ORs in macrophages and their potential role in inflammation and disease. We will also discuss how these findings can be translated into possible therapeutic applications.

## Olfactory receptor expression in macrophages

The first evidence that ORs can be expressed in macrophages was made in 2013 by Li et al. in the lung and airways, identified by Illumina microarray technology (15). They described the expression of several ORs already at a steady state. Some of them were also confirmed by real time quantitative polymerase chain reaction (RTqPCR) (15). Interestingly, the expression of at least eight Oss (ORL1014 known as Or6b2, ORL657, ORL622, ORL568 known as Or8g18, ORL446 known as Or8u8, ORL352, ORL272, and ORL65) were significantly upregulated by the coincubation of lipopolysaccharide (LPS) and IFNγ (15). ORL622 expression was ≈5 times upregulated compared to LPS or IFNγ alone. This was confirmed at the protein level by immunofluorescence in pulmonary macrophages. ORs recently received new, systematic gene names, which are found in in the Olfactory Receptor Database (https://senselab.med.yale.edu/ORDB/).

Our group recently described that LPS alone was able to boost the RNA expression of *Olfr2* in bone marrow–derived macrophages (BMDMs) (13). However, the mechanism was not identified. We detected both mouse Olfr2 and human OR6A2 in a vesicular compartment of macrophages, using immunofluorescence and confocal microscopy. LPS stimulation and the stimulation with the specific ligand octanal-mediated trafficking of OR6A2 and Olfr2 from the vesicular compartment to the plasma membrane. LPS and octanal also increased the mRNA for *Olfr2* and *OR6A2* as measured by RTqPCR.

*Olfr2* and *Olfr78* expression was also detected and confirmed in BMDMs (13, 14). *In vivo*, vascular macrophages from mice challenged for 2, 4, 8, or 12 weeks with a western diet (WD, 42% of calories from fat) expressed *Olfr2*. Macrophages from WT mice also expressed *Olfr2*; however, the WD boosted its expression. It is likely that human vascular macrophages express the orthologue *OR6A2* based on the immunofluorescence of excised aortic tissue and on mRNA expression in the Biobank of Karolinska Endarterectomies (BIKE) dataset that includes the gene expression analysis (Affymetrix chip) of more than 120 patients undergoing endarterectomy operations (GSE21545). The human plaques of the BIKE dataset have macrophage content ranges between 35% and 65% as defined by CIBERSORT (13). We found that *OR6A2* expression is positively correlated with the macrophage content (13). These data support the idea that OR6A2 may contribute to atherosclerosis in humans. OR6A2 mRNA and protein expression were also described in human monocyte–derived macrophages (hMDMs) (13).

**Figure 1:**
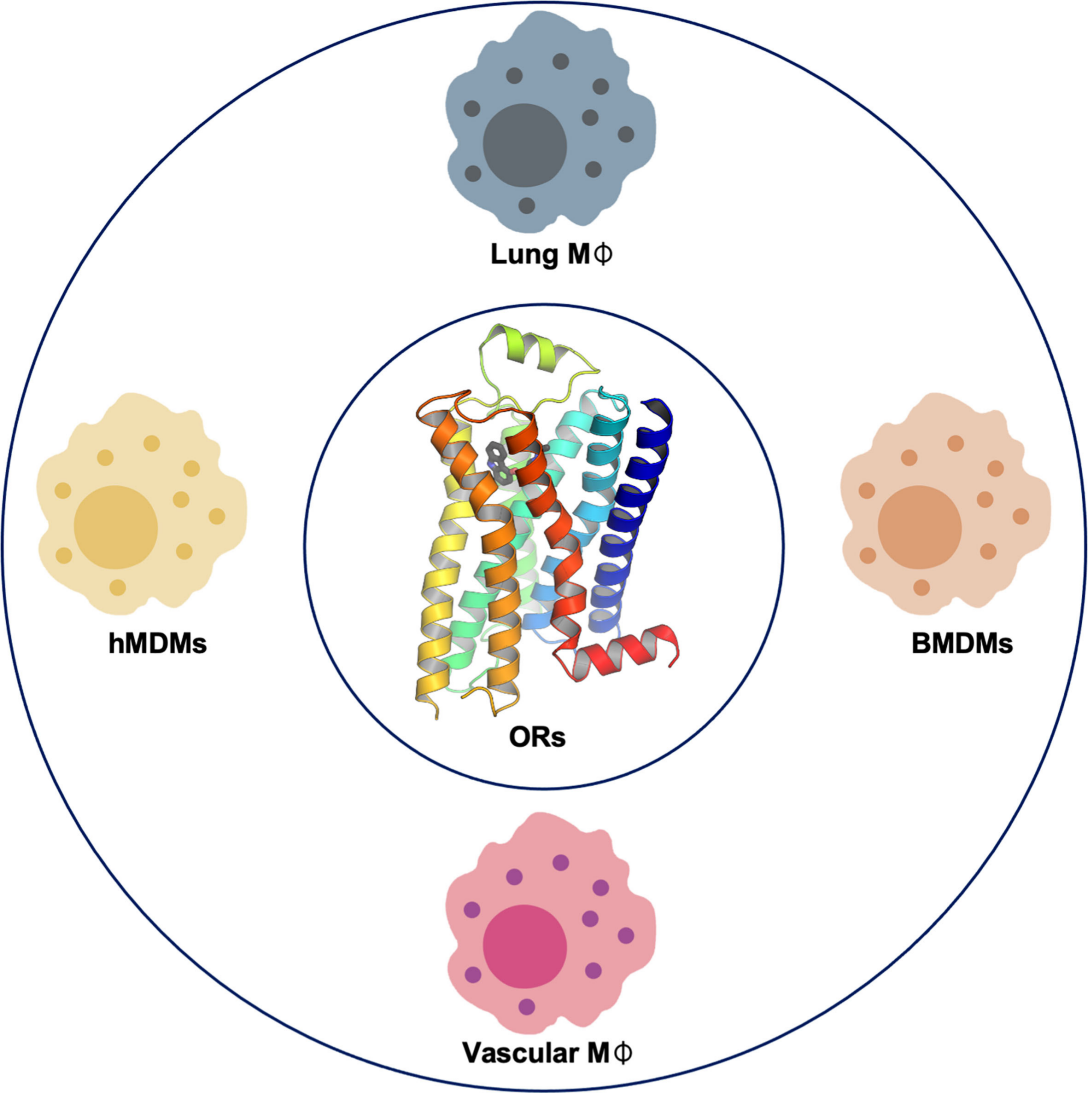
Olfactory receptor (OR) confirmed gene and protein expression in macrophages. Gene and protein expression of specific ORs have been reported and confirmed in lung macrophages (15), bone marrow–derived macrophages (BMDMs) (13, 14), and vascular macrophages in both human and mice and human monocyte–derived macrophages (hMDMs) (13).

Confirmed OR expression in macrophages at the mRNA and protein levels is summarized in **Figure 1**.

**Figure 2:**
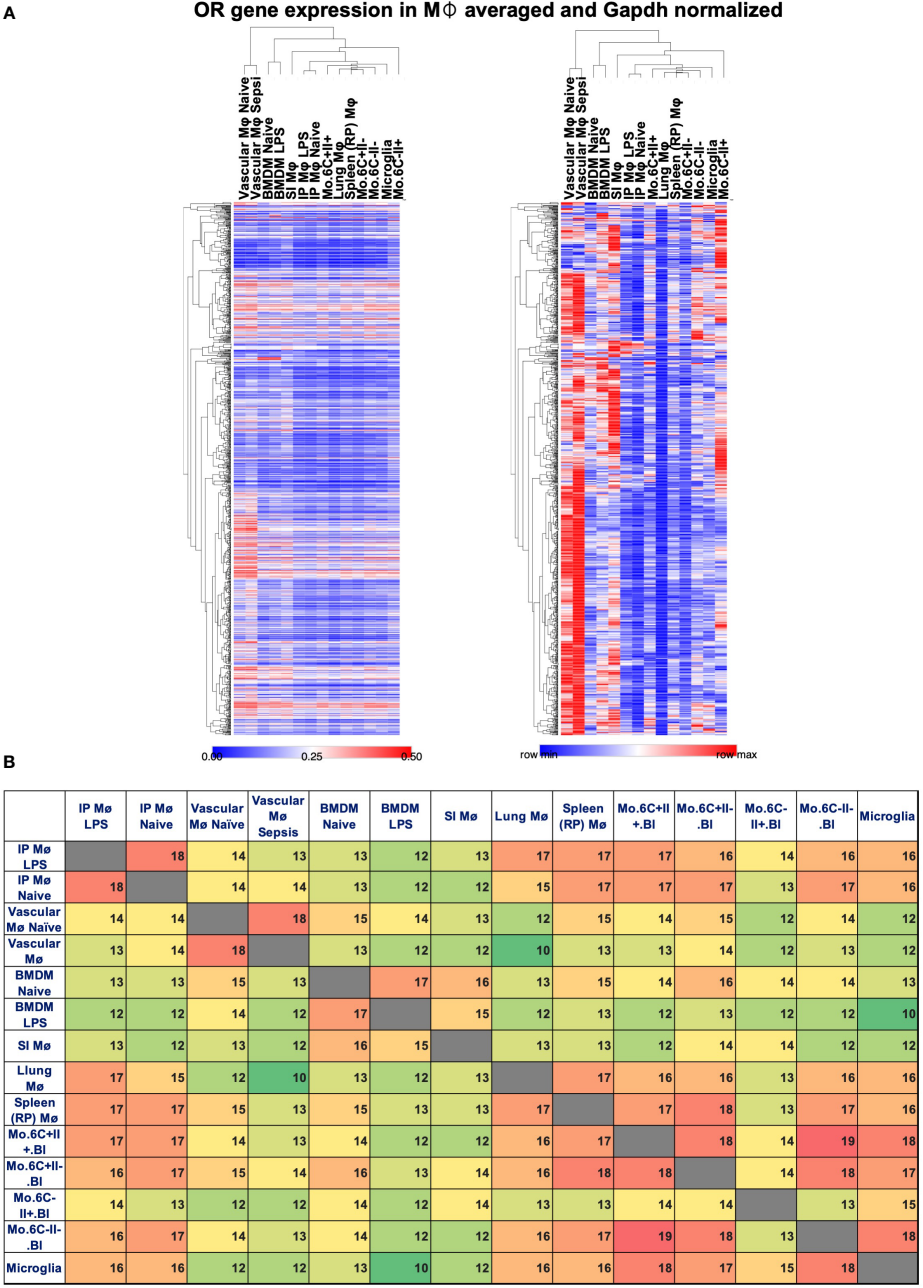
OR expressed by gene in macrophages and monocytes. Several ORs identified by gene expression analysis in macrophages and monocyte datasets. **(A)** Heat maps reporting the relative Gapdh-normalized expression (left, scale 0–0.5) or row min/row max (right) in vascular macrophages (Mø), BMDMs, intraperitoneal (IP) macrophages treated or untreated with LPS, small intestine (SI) Mø, red pulp (RP) Mø, lung CD11b^-^ Mø, and monocyte (mo.) subsets defined based on the expression of LY6C (6C) and MHCII (II). One minus Pearson correlation clustering of rows and columns. **(B)** Pairwise intersection analysis of top 20 expressed ORs in the datasets, color scaled green to red based on low-to-high coexpression.

To look for possible OR expression in other mouse tissue macrophages and monocytes, we analyzed several published datasets (Affymetrix mouse gene 1.0 ST array type): GSE68968 Vascular Mφ; GSE77104 BMDMs, GSE43075 intra-peritoneal (IP) Mφ; GSE100393 Small Intestine (SI) Mφ; GSE15907 Lung Mφ; GSE15907 Spleen (Red pulp) Mφ; GSE15907 Microglia; and GSE15907 blood classical (CM) and non-classical monocyte (NCM) subpopulations. Affymetrix gene chip data were normalized using the robust multiarray average (RMA) method (log2, background-corrected, quantile- normalized). The average values among the respective replicates were used. Pearson correlation was then applied to compare gene expression among datasets, normalized by *Gapdh* expression (Xmax) using the ranking strategy: (X-Xmin)/(Xmax-Xmin) with "X" being the respective OR expression level and "Xmin" the minimum level of OR expression per dataset. Heatmap analysis and visualization were performed by Morpheus, https://software.broadinstitute.org/morpheus. This analysis shows that other tissue macrophages and monocytes also express mRNA for some ORs, but the subsets are different by the tissue source and the monocyte type. The level of expression also varies (**Figure 2A**). Vascular Mφ shows a higher level of normalized OR expression, whereas naïve IP Mφ and lung Mφ show lower levels of OR expression among the subsets (**Figure 2A**). The list of *Gapdh*-normalized OR expression can be found in **Supplementary Tables 1a, b**. Analyzing the top 20 expressed ORs for each macrophage and monocyte subset, we found that the expression of several OR genes are shared among all datasets such as *Olfr239, Olfr314, Olfr401, Olfr462, Olfr71, Olfr91, Olfr982*, and the pseudogene *Olfr1174-ps* (**Supplementary Tables 2a, b**). Only a few are exclusive, such as *Olfr1502* and *Olfr147* in naïve vascular Mφ; *Olfr109* in naive BMDMs; *Olfr166*, *Olfr63*, and *Olfr984* in LPS-treated BMDMs; *Olfr267* in SI Mφ; and *Olfr444* and *Olfr328* in LY6C+ MHCII- monocytes (**Supplementary Tables 2a, b**). Interestingly, the pairwise intersection analysis of the top 20 expressed ORs shows how the monocyte and macrophage subsets maintain a high similarity even after LPS treatment (**Figure 2B**). The expression pattern of ORs is largely specific to the tissue macrophage or monocyte subpopulations (**Figure 2B**). LY6C-MHCII+ monocytes, for example, share the least amount of top 20 ORs with other macrophage and monocyte populations (**Figure 2B**).

In summary, ORs can be differentially expressed in specific tissue macrophages and monocyte populations (**Figure 2**). However, the functionality of most of these ORs remains unclear at this time.

## Olfactory receptor signaling and function in macrophages

Li et al. were the first to report possible functions played by ORs in macrophages. Their initial exploration suggested that octanal (10 µM) was able to boost LPS-induced CCL2 (MCP-1) expression that was decreased by diltiazem (a Ca^2+^ ion channel blocker) (30). Diltiazem is a well-established drug known to inhibit CNG channels and block the OR function in the OE (15, 31). Li et al. suggested an increased macrophage migration measured by the transwell migration assay in response to supernatants derived from macrophages treated with IFNγ/LPS and octanal as compared to IFNγ/LPS only (15). They did not find any changes in bacterial uptake or macrophage polarization. Possible disease relevance was not investigated.

Recently, we found that the octanal receptor Olfr2 can serve as signal 2 and activate the NLRP3 inflammasome, driving IL-1β and IL-1α release (13). Inflammasome activation requires signal 1 (gene expression) and signal 2 (activation) (32). We identified that Olfr2 signaling in macrophages was likely to follow the known signaling cascade described in the OE. In the OE, ORs couple through Golf. Upon ligand binding, Golf activates Adcy3, which produces cAMP that, in turn, triggers CNG calcium channels (i.e., CNGA 1 and 3) (10). We confirmed that the genes encoding olfactory signal transduction such as Adcy3 and the cation channels CNGA1,2,3,4 and CNGB1 were expressed in vascular macrophages. Octanal, a cognate ligand for Olfr2, induced cAMP elevation and a Ca^2+^ flux with kinetics consistent with CNG channels. BMDMs from *Adcy3*+/- mice showed a reduced calcium flux. The CNG blocker diltiazem completely abrogated the calcium flux and IL-1 production.

How exactly Olfr2 ligation produces signal 2 remains unclear. A Ca^2+^ flux has been proposed as a signal that is able to activate the inflammasome assembly after priming (33), but this is controversial (34). We showed that octanal-treated macrophages differentially express genes involved in oxidative stress pathways and ROS production. To test whether octanal, through Olfr2, Adcy3, and a Ca^2+^ flux induces ROS production for signal 2, we measured cellular and mitochondrial ROS and found both elevated after Olfr2 ligation (13). This suggests that ROS may be responsible for Nlrp3 inflammasome activation leading to IL-1 secretion in response to octanal (13). Human OR6A2 in MDMs was also shown to respond to octanal and activate the NLRP3 inflammasome suggesting similar functionality as Olfr2 in mouse macrophages (13).

**Figure 3:**
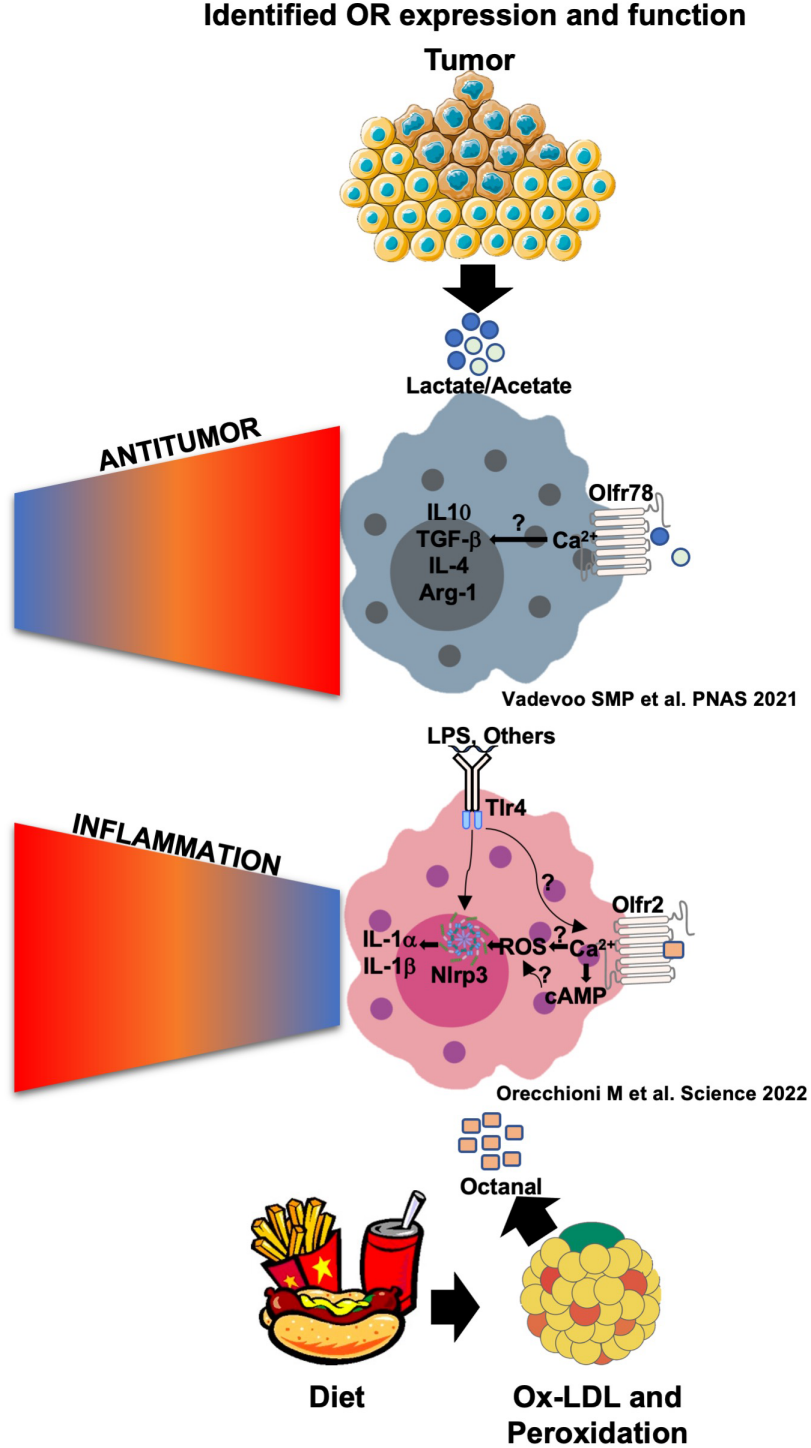
Olfr78 and Olfr2 signaling and function in response to their ligands in macrophages. Olfr78 has been reported to mediate the upregulation of the mRNA levels of M2-like cytokines such as IL-4, IL-10, TGF-β, and Arg1 in response to lactate that is highly abundant in the tumor microenvironment. This promotes immunosuppressive functions and tumor progression. Olfr78 signaling mechanisms in macrophages remain unknown. Olfr2 is ligated by octanal, which is likely derived from lipid peroxidation. Oxidized low-density lipoprotein (oxLDL) contains enough octanal to activate Olfr2, and this activation is gone in Olfr2 knockout macrophages. Olfr2 ligation mediates an increase in cAMP, which activates CNG-type calcium channels. The resulting Ca^2+^ influx causes increased reactive oxygen species (ROS), which serve as signal 2 for NLRP3 inflammasome activation. Olfr2 ligation is proinflammatory and exacerbates atherosclerosis.

To test disease relevance, we used the *Ldlr^-/-^* mouse model of atherosclerosis (35). In this model, feeding mice a high-cholesterol diet leads to atherosclerotic lesions in the aorta and the aortic root. We performed several bone marrow transfer experiments. *Olfr2^-/-^* bone marrow cells were transferred to lethally irradiated *Ldlr^-/-^* recipient mice and set on a high-cholesterol diet. After 8 weeks, mice were injected IP every other day with octanal (10 µg/g) or a vehicle for four extra weeks for a total of 12 injections. *Ldlr^-/-^* mice reconstituted with WT bone marrow and treated with octanal showed a significant average increase (≈8%) of *en face* lesions compared to the vehicle-treated group (≈6%). By contrast, in *Ldlr^-/-^* mice reconstituted with *Olfr2^-/-^* BM, the average lesion size was only ≈2% and was not changed by octanal treatment. Aortic root lesions by serial sections were also reduced in mice that had received the *Olfr2^-/-^* bone marrow. We concluded that boosting octanal levels in mice exacerbates and knocking out Olfr2 significantly reduces atherosclerosis progression in mice. These findings show that Olfr2 is involved in exacerbating atherosclerosis. Since knocking out Olfr2 reduces atherosclerosis, Olfr2 and, by extension, OR6A2, are potential therapeutic targets for the prevention and treatment of atherosclerosis (**Figure 3**).

In 2021, Vadevoo and colleagues explored the functionality of another OR called Olfr78 (14) previously described to be expressed in kidneys and other organs (36) and to be relevant for renin secretion in response to short-chain fatty acids (SCFAs) (37, 38). They found that Olfr78 is expressed in at least 40% of BMDMs at a steady state. Olfr78 expression increased in BMDMs differentiated to a M2-like phenotype by IL-4 incubation, and similar results have been found by incubating BMDMs with tumor-conditioned media and lactate (10 mM) (14). Lactate has been shown to activate Olfr78. The concentration of lactate in the tumor-conditioned media (TCM) of human and mouse tumors is ∼8–20 mM and has been reported to affect many cell types, including macrophage differentiation, promoting protumoral M2-TAMs mediating immunomodulation and tumor progression (39). The stimulation of BMDMs with LPS and IFNγ downregulates Olfr78 expression in BMDMs. Thus, the regulation of Olfr78 in macrophages is clearly different from that of Olfr2.

Lactate as well as TCM has been found to mediate the expression and secretion of several M2-like cytokines such as IL-4, IL-10, and TGFβ. This increase was gone in *Olfr78^-/-^* macrophages (14). To test whether lactate is the main factor in TCM responsible for Olfr78-mediated activation, the authors used the lactate inhibitor oxamic acid, successfully depleting lactate in TCM. Lactate-depleted TCM was less able to mediate M2 macrophage polarization and IL-4, IL-10, and TGFβ secretion. Intriguingly, Olfr78 depletion improved survival in a mouse model of Lewis lung carcinoma (referred to from now on as “lung tumor”). Similarly, tumor growth was significantly slower in *Olfr78^-/-^* mice compared to wild-type mice. The authors also suggested that CD45+Ly6C-MHCII+ M1 macrophages, as well as CD8+ T cells and CD4+ T cells, increased in lung tumors implanted into *Olfr78^-/-^* mice (**Figure 3**). The human Olfr78 ortholog OR51E2 is also known to bind SCFAs and is highly expressed in prostate cancer and possibly other cancers (6, 40, 41). Lower OR51E2 expression is significantly correlated with longer survival in Kaplan–Meier analysis (14).

The overall signaling pathway of Olfr78 in response to lactate as well as the mechanism for the observed modulation in macrophages has not been described in the manuscript (14). The possible differences found in the functionality of the so-far characterized OR may be due to different signaling pathways involved such as possible different G proteins and will require more extensive investigations in the future.

## Olfactory receptor deorphanization strategies in macrophages

ORs are starting to be evaluated for therapeutic potential and physiological importance. In total, 80% of human ORs have no known ligands and are referred to as “orphan” receptors. Deorphanization, which means the pairing of receptors with respective ligands, is thus an important objective in this field. Several cell lines including HeLa and Hana3A can be used for OR deorphanization purposes (42). Exogenous food odorants such as SCFAs as well as natural or synthetic odorant compounds and endogenous metabolites are reported to activate distinct OR in these *in vitro* cell lines (43). So far, the ORs expressed in macrophages have been linked with endogenous oxidation and metabolic processes that modulate possible inflammatory and disease states. However, several more receptors are potentially expressed and very few have known ligands. The deorphanization of ORs expressed in macrophages is becoming crucially important for the identification of their function.

The strategies used in previously reported cell lines can be also applied in primary macrophages or macrophage cell lines (44). Because we know that some ORs expressed in macrophages are functional, the direct use of them might decrease the difficulty of expressing functional exogenous ORs as the cellular machinery for their function is already able to process OR signaling. A limitation could be a readout. The singling pathway in macrophages as well as in other ectopic tissues is still unclear for many newly identified ORs. New experimental evidence suggests that a Ca^2+^ flux seems always required for the functionality of ORs expressed in macrophages and can be used as a readout (13, 14).

Targeting specific OR expression by using gene-editing strategies such as small- interference RNAse (siRNA) knockdown or CAS9-mediated knockout in BMDMs or macrophage cell lines followed by treatments with candidate odorants using Ca^2+^ as a readout is a possible approach for ligand identification. Because some ORs including mouse Olfr2 and human OR6A2 mediate the activation of the NLRP3 inflammasome, inflammasome products such as IL-1β, IL-1α, and IL-18 could be used as more distal readouts. Such experiments would also give an indication of the proinflammatory function of the studied receptor.

## Future perspective and conclusions

The scientific community is starting to accept that ORs have important roles beyond the nose (3). ORs have been implicated in the regeneration of keratinocytes (45), sperm motility (5), the control of blood pressure (46, 47), cancer and atherosclerosis, and more (8, 9). ORs have also been suggested to modulate tumor progression, such as in acute and chronic myelogenous leukemia (48). Some metabolites derived from diet, metabolism, microbiota, oxidative stress, and inflammation may be OR ligands and impact several human diseases (49, 50).

Approximately 40% of all pharmaceutical drugs on the market target GPCRs (51). ORs are the largest group within the mammalian GPCR gene superfamily. Their functional exploration outside the nose and specifically in macrophages is in its infancy, yet many ORs are expressed in macrophages, most with unknown function. It appears that macrophages and monocytes express several types of ORs as sensors for small molecules in their environment (26). After sensing perturbations, macrophages can produce many molecules such as cytokines modulating inflammation and disease (52). One reasonable hypothesis is that ORs change gene expression in macrophages based on the activation status as well as diet or disease. ORs can indeed sense metabolites present in the blood and tissues, possibly playing a role in controlling homeostasis or responding to stress and disease.

The current limitation in understanding OR function in macrophages is the lack of characterization of most ORs and the high amount of orphan receptors that do not have a known ligand. Thus, OR deorphanization is a crucial step for the exploration of OR functionality in macrophages and monocytes. Targeting ORs expressed in macrophages and monocytes may constitute novel therapeutic targets for the treatment, prevention, and reversal of diseases including atherosclerosis and cancer.

## Author contributions

KL, HM, and MO wrote the paper. MO performed the analysis, constructed the figures, and gave input to the **Supplementary Tables**. All authors contributed to the article and approved the submitted version.

## Funding

This study was funded by the National Institutes of Health R01 HL115232 (to KL), DC016224 and DC020353 (to HM), and the American Heart Association Career Development Award AHA Award Number: 941152 (to MO).

## Conflict of interest

MO and KL are named as coinventors on US Patent App. 17/048,059 held by the La Jolla Institute for Immunology relating to cardiovascular diagnostics and therapeutics and may receive royalty payments for inventions or discoveries related to cardiovascular diagnostics or therapeutics. HM has received royalties from ChemCom. HM has received research grants from Givaudan. HM has received consultant fees from Kao.

## Publisher’s note

All claims expressed in this article are solely those of the authors and do not necessarily represent those of their affiliated organizations, or those of the publisher, the editors and the reviewers. Any product that may be evaluated in this article, or claim that may be made by its manufacturer, is not guaranteed or endorsed by the publisher.
